# Differential astrocyte and oligodendrocyte vulnerability in murine Creutzfeldt-Jakob disease

**DOI:** 10.1080/19336896.2021.1935105

**Published:** 2021-07-05

**Authors:** Pol Andrés-Benito, Margarita Carmona, Jean Yves Douet, Hervé Cassard, Olivier Andreoletti, Isidro Ferrer

**Affiliations:** a Department of Pathology and Experimental Therapeutics, University of Barcelona; Biomedical Research Centre of Neurodegenerative Diseases (CIBERNED), Institute of Health Carlos III, Ministry of Economy, Innovation and Competitiveness, Hospitalet De Llobregat; Bellvitge Institute of Biomedical Research (IDIBELL); Institute of Neurosciences, University of Barcelona, Barcelona; Spain; b Interactions Hôte Agent Pathogène , UMR INRA ENVT 1225-IHAP, École Nationale Vétérinaire De Toulouse, Toulouse, France

**Keywords:** Prionopathy, creutzdfeldt-jakob, astrocytes, oligodendrocytes, myelin

## Abstract

Glial vulnerability to prions is assessed in murine Creutzfeldt-Jakob disease (CJD) using the tg340 mouse line expressing four-fold human PrP M129 levels on a mouse PrP null background at different days following intracerebral inoculation of sCJD MM1 brain tissues homogenates. The mRNA expression of several astrocyte markers, including glial fibrillary acidic protein (*gfap*), aquaporin-4 (*aqp4*), solute carrier family 16, member 4 (*mct4*), mitochondrial pyruvate carrier 1 (*mpc1*) and solute carrier family 1, member 2 (glial high-affinity glutamate transporter, *slc1a2*) increases at 120 and 180 dpi. In contrast, the mRNA expression of oligodendrocyte and myelin markers oligodendrocyte transcription factor 1 (*olig1), olig2*, neural/glial antigen 2 (*cspg*), solute carrier family 16, member 1 (*mct1*), myelin basic protein (*mbp*), myelin oligodendrocyte glycoprotein (*mog*) and proteolipid protein 1 (*plp1*) is preserved. Yet, myelin regulatory factor (*myrf*) mRNA is increased at 180 dpi. In the striatum, a non-significant increase in the number of GFAP-positive astrocytes and Iba1-immunoreactive microglia occurs at 160 dpi; a significant increase in the number of astrocytes and microglia, and a significant reduction in the number of Olig2-immunoreactive oligodendrocytes occur at 180 dpi. A decrease of MBP, but not PLP1, immunoreactivity is also observed in the striatal fascicles. These observations confirm the vulnerability and the reactive responses of astrocytes, together with the microgliosis at middle stages of prion diseases. More importantly, these findings show oligodendrocyte vulnerability and myelin alterations at advanced stages of murine CJD. They confirm oligodendrocyte involvement in the pathogenesis of CJD.

## Introduction

Prion diseases are fatal neurodegenerative diseases linked to the transformation of normal prion protein (PrP^C^) into an abnormal prion protein (PrP^Res^) which is transmitted from one cell to another causing neuron loss, astrocytic gliosis, microglia activation, and, frequently, spongiform change. This transformation leads to devastating effects on the central nervous system including abnormal behaviour in animals, and cognitive impairment and rapid dementia in humans. Creutzfeldt-Jakob disease (CJD) and scrapie are the most common natural paradigms of sporadic prionopathies in humans, and sheep and goats, respectively [1–4]. CJD can be categorized depending on the prion type and the phenotype of codon 129 in *PRNP*; sporadic CJD methionine/methionine type 1 (sCJD MM1) is the most common form of CJD [5,6].

PrP^C^ modulates the differentiation of stem-cells to neurons, astrocytes, and oligodendrocytes during development [7,8]. In contrast to other neural cell types, oligodendrocytes are considered resistant to prions [9]. However, a putative trans-membrane domain of the human PrP^C^ induces apoptosis of oligodendrocytes *in vitro* in a time- and dose-dependent manner [10]. PrP^Res^ immunoreactivity is observed as arrays adjacent to myelinated fibres and as clumps adjacent to oligodendroglial nuclei in the cerebrum and cerebellum in sCJD [11]. Large, PrP^Res^-immunoreactive perinuclear and nuclear deposits may occur in oligodendrocytes in a few cases with sCJD [12]. Morphological observations are complemented by biochemical studies showing reduced expression of a few oligodendrocyte cell markers in the cerebral cortex at the terminal stages of sCJD [13]. The present study was designed to assess the involvement of oligodendrocytes and astrocytes at preclinical and clinical stages in tg340 mice inoculated with human brain homogenates of sCJD. The study was aimed at gaining information about oligodendrocyte and astrocyte vulnerability at preclinical stages and with disease progression in a validated model of experimental CJD.

## Material and methods

The tg340 mouse line expressing about four-fold level of human PrP M129 on a mouse PrP null background was generated as described elsewhere [14]. Inocula were prepared from sCJD MM1 brain tissues as 10% (w/v) homogenates. Mice aged 6–10 weeks old were anaesthetised and inoculated in the frontal cortex using a 25-gauge disposable hypodermic needle, as detailed elsewhere [15] (four-six animals per group and time point). Mice were observed daily and the neurological status was assessed weekly. Mice were euthanized at 0, 60, 120 (pre-clinical), 160, and 180 (clinical) days post-inoculation (dpi), necropsy was performed, and the brain was rapidly removed. One hemisphere was fixed by immersion in 4% buffered formalin; the other was frozen at −80°C and stored at this temperature until use. The samples fixed in buffered formalin were then transferred to a solution of formic acid, washed, and embedded in paraffin. Unfortunately, not all samples were available for biochemical and morphological studies due to unexpected accidental events. Biochemical studies were limited to mRNA expression as not enough material was available for western blot studies on account of the same reasons. All animal experiments were performed in compliance with institutional and French national guidelines in accordance with the European Union Directives 86/609/EEC and 2010/63/EU. Experiments were approved by the Committee on the Ethics of Animal Experiments of the author’s institutions: INRA Toulouse/ENVT (Permit Number: 01734.01).

Gene expression was analysed at 120, 160, and 180 dpi, and corresponding age-matched controls; morphology and immunohistochemistry at 0, 60, 120, and 180 dpi, and corresponding controls. We used the miRvana isolation kit (Ambion, USA) to obtain RNA of sCJD MM1 mice and controls. Then, samples were treated with RNase-free DNase set (Ambion, US) for 30 min to avoid the extraction and subsequent amplification of genomic DNA. The concentration of each sample was determined at 340 nm using NanoDrop 2000 spectrophotometer (Thermo Scientific, USA). High Capacity cDNA Archive kit (Applied Biosystems, US) following the protocol provided by the manufacturer and using the Gene Amp® 9700 PCR System thermocycler (Applied Biosystems, USA) was used for the retrotranscriptase reaction of RNA samples. A parallel reaction for an RNA sample was run in the absence of reverse transcriptase to assess the degree of contaminating genomic DNA. PCR assays were conducted in duplicate on cDNA samples obtained from the retrotranscription reaction and diluted 1:15 in 384-well optical plates (Applied Biosystems, USA) utilizing an ABI Prism 7900 Sequence Detection System (Applied Biosystems, USA). Parallel amplification reactions for each sample were carried out using the 20× TaqMan Gene Expression Assays (Applied Biosystems, USA) and 2× TaqMan Universal PCR Master Mix (Applied Biosystems, USA). We used the internal housekeeping genes alanyl-tRNA synthetase (*aars*), β-glucuronidase (*gusβ*), and X-prolyl aminopeptidase P1 (*xpnpep1*) for normalization. Probes used in this study are shown in Table 1. The reactions were performed as follows: 50°C for 2 min, 95°C for 10 min, and 40 cycles of 95°C for 15 s, and 60°C for 1 min. TaqMan PCR data were captured using the Sequence Detector Software (SDS version 2.1, Applied Biosystems, USA). We used the double-delta cycle threshold (ΔΔCT) method to analyse the results. ΔΔCT values were calculated as the ΔCT of each test sample minus the mean ΔCT of the calibrator samples for each target gene. The fold change was determined using the equation 2(−ΔΔCT).

**Table 1.** List of taqman probes used in gene expression studies

Gene		Full name	Reference
*aars*	alanyl-tRNA synthetase		Mm00507627_m1
*aqp4*	Aquaporin-4		Mm00802131_m1
*cspg4*	Neural/glial antigen 2		Mm00507257_m1
*gfap*	Glial fibrillary acidic protein		Mm01253033_m1
*gus-β*	β-glucuronidase		Mm01197698_m1
*mbp*	Myelin Basic Protein		Mm01266402_m1
*mct1*	Solute Carrier Family 16 (Monocarboxylic Acid Transporters), Member 1		Mm01306379_m1
*mct4*	Solute Carrier Family 16 (Monocarboxylic Acid Transporters), Member 4		Mm01246825_m1
*mog*	Myelin Oligodendrocyte Glycoprotein		Mm01279062_m1
*mpc1*	Mitochondrial Pyruvate Carrier 1		Mm01316203_g1
*myrf*	Myelin Regulatory Factor		Mm01194959_m1
*olig1*	Oligodendrocyte Transcription Factor 1		Mm00497537_s1
*olig2*	Oligodendrocyte Lineage Transcription Factor 2		Mm01210556_m1
*plp1*	Proteolipid Protein 1		Mm01297210_m1
*slc1a2*	Solute Carrier Family 1 (Glial High Affinity Glutamate Transporter), Member 2		Mm01275814_m1
*xpnpep1*	X-prolyl aminopeptidase (aminopeptidase P) 1		Mm00460030_m1

Formalin-fixed, formic acid-treated, paraffin-embedded de-waxed sections, 4 μm thick, were stained with haematoxylin and eosin, or processed for immunohistochemistry. The sections were boiled in citrate buffer (20 min). Endogenous peroxidases were blocked by incubation in 10% methanol-1% H_2_O_2_ solution (15 min) followed by 3% normal horse serum solution. Then, the sections were incubated at 4°C overnight with one of the primary antibodies against glial fibrillary acidic protein (GFAP) (rabbit polyclonal, used at 1:500, Dako, Glostrup, DK), Olig-2 (rabbit polyclonal, used at 1:500, Abcam, Cambridge, UK), Iba1 (rabbit polyclonal, used at 1:1,000, Wako, Osaka, Japan), MBP (rabbit polyclonal, used at 1:500, Dako, Glostrup, DK), PLP1 (mouse monoclonal, used at 1:100, LSBio, Seattle, WA, USA) and PrP (mouse monoclonal, used at 1:50, BioReagents Tech., France) with and without pre-incubation with proteinase K. Following incubation with the primary antibody, the sections were incubated with EnVision + system peroxidase (Dako, DK) for 30 min at room temperature. The peroxidase reaction was visualized with diaminobenzidine and H_2_O_2_. Control of the immunostaining included omission of the primary antibody; no signal was obtained following incubation with only the secondary antibody. Peptides for pre-absorption studies were not available.

Quantifications of GFAP‐, Olig2‐, and Iba1‐immunoreactive cells in the anterior striatum were made by counting the number of positive cells in three areas per section of the anterior striatum selected at random measuring 0.05 mm^2^ each, one section per mouse, and all mice at the different dpi using a DP25 camera adapted to an Olympus BX50 light microscope. The number of positive cells was counted directly on the figures and expressed as the number of positive cells per area. The density of MBP was calculated in three areas per section of the anterior striatum selected at random measuring 0.162 mm^2^ each, one section per mouse, and for all mice at the different dpi. Densitometry was calculated using the Analysis tool of the Adobe® Photoshop® CS5 software (Adobe Systems Inc., San Jose, CA, USA). The density of MBP was calculated as the intensity of brown colour normalized for the total area excluding white spaces of the nuclei and expressed as arbitrary units per area. Quantifications and densitometry were performed by a researcher blind to the corresponding MM1-inoculation or incubation time of each section.

Statistical analysis was performed with the SPSS Statistics v21.0 software (IBM Corp. Released 2013, IBM-SPSS Statistics for Windows, Version 21.0., Armonk, NY, USA). The normality of distribution was analysed with the Kolmogorov–Smirnov test. Gene expression data were analysed with two-way analysis of variance (ANOVA) with MM1-inoculation and incubation time as between factors, followed by Bonferroni’s *post hoc* test when required. The data were expressed as mean ± SEM. Differences between the control group and its respective inoculated group were considered statistically significant at *p < 0.05, **p < 0.01, ***p < 0.001.

Densitometry and quantification data were analysed with one‐way ANOVA followed by Tukey’s post-hoc to compare inoculum incubation times. The data were expressed as mean ± SEM. Differences between inoculated groups at different incubation times were set at **p < 0.01, ***p < 0.001 vs. 0 dpi; ## p < 0.01, ### p < 0.001 vs. 60 dpi; and $ p < 0.05, $$$ p < 0.001 vs. 120 dpi. Outliers were detected using the GraphPad software QuickCalcs (p < 0.05). Graphic design was made with GraphPad Prism version 5.01 (La Jolla, CA, USA).

## Results

**Figure 1.**
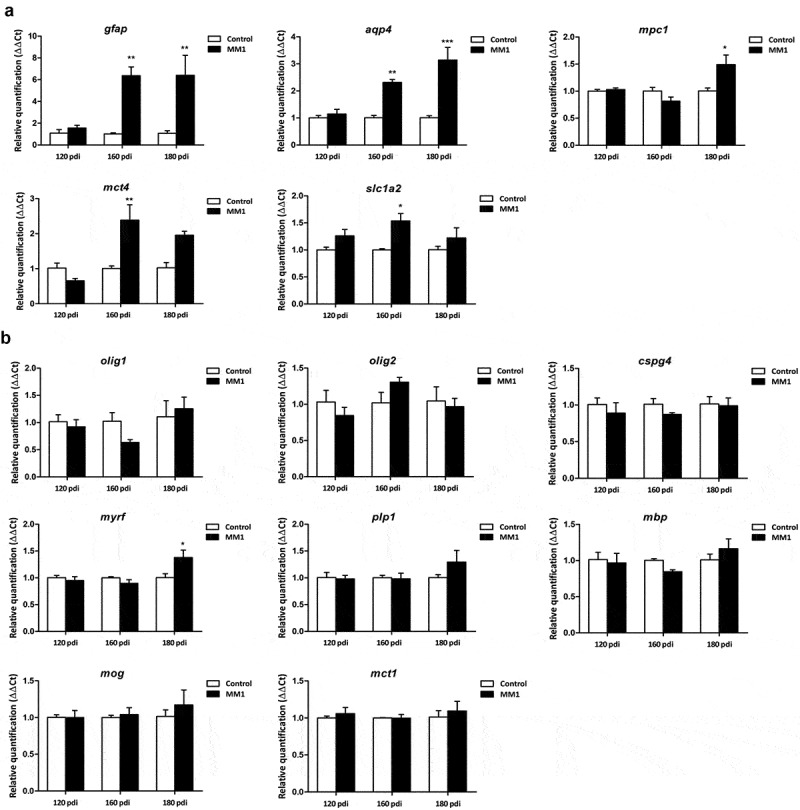
Expression levels, as revealed by qPCR, of genes coding for specific proteins of astrocytes (a) and oligodendrocytes (b) in the cerebrum of tg340 control and tg340 sCJD MM1-inoculated mice at 0, 60, 120 (preclinical), and 180 (clinical stage) days post-inoculation (dpi). A significant increase in the expression of *gfap* and *aqu4* is found at 160 and 180 dpi, whereas levels of *mpc1*, coding for mitochondrial pyruvate carrier 1, at 180 dpi, and *mct4* and *slc1a2*, coding for monocarboxylic acid transporter member 4, and glial high affinity glutamate transporter, respectively, were significantly increased at 160 dpi with a trend not reaching statistical significance at 180 dpi. In contrast, expression levels of *olig1, cspg4* (coding for NG2), *plp1, mbp*, and *mog* (coding for proteolipid protein 1, myelin basic protein, and myelin oligodendrocyte glycoprotein, respectively), did not show modifications up to 180 dpi. The levels of *mct1* (coding for monocarboxylic acid transporter 1) were not affected. Only *myrf* was significantly increased at 180 dpi. Differences are considered statistically significant at * p < 0.05, ** p < 0.01, *** p < 0.001

Two-way ANOVA revealed a significant interaction between sCJD MM1-inoculation and the progression of the incubation time for *aqp4* [F [2,16] = 6.960 (P = 0.0058)], *mpc1* [F [2,16] = 5.302 (P = 0.0154)], and *mct4* [F [2,15] = 5.066 (P = 0.02)] gene expression (Figure 1a). Bonferroni’s *post hoc* test showed significant differences between mice inoculated with human CJD MM1 homogenates and age-matched controls. Increased levels of *aqp4* were found in inoculated animals at 160 dpi (P < 0.01) and 180 dpi (P < 0.000) when compared with age-matched controls. Similarly, significantly high levels of *mpc1* mRNA were found in inoculated animals at 180 dpi (P < 0.05) when compared with age-matched non-inoculated mice. Finally, high levels of *mct4* were found in inoculated mice at 160 dpi (P < 0.01) when compared with the corresponding control group.

**Figure 2.**
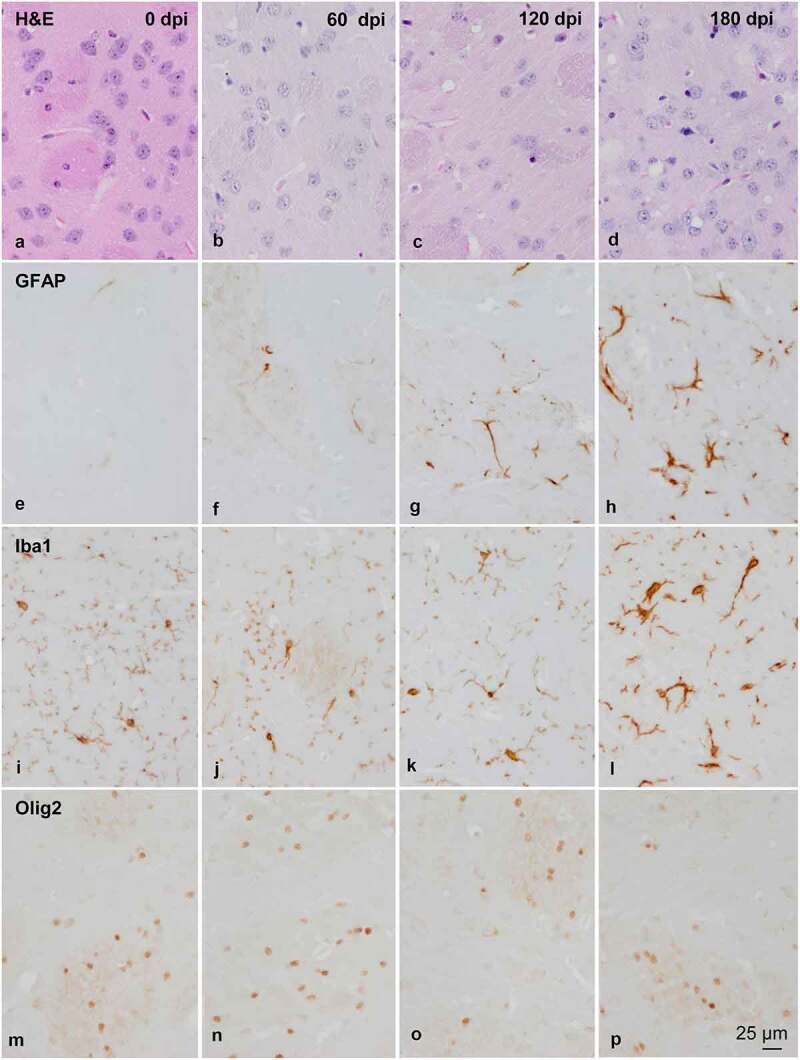
Morphology and immunohistochemistry of the striatum in tg340 control and tg340 sCJD MM1-inoculated mice at 0, 60, 120 (preclinical), and 180 (clinical stage) days post-inoculation (dpi). A few vacuoles consistent with spongiform change are first seen in haematoxylin and eosin (H&E) stained sections at 120 dpi, and their number increases at 180 dpi (a-d). This is accompanied by a moderate, non-significant increase, in the number of reactive astrocytes and microglia at 120 dpi, and with marked increase of GFAP-immunoreactive cells (e-h) and microglia (Iba1-positive cells) (i-l) at 180 dpi. The number of oligodendrocytes, as revealed with the Olig2 antibody, shows a trend to decrease in the intrastriatal fibres at 180 dpi (m-p). The immunoreactivity to proteolipid protein 1 (PLP1) is preserved at 180 dpi (q-t). Nevertheless, some intrastriatal fascicles show focal, cotton-like decrease of myelin basic protein (MBP) immunoreactivity at the same time-point (u-y). These alterations occur in parallel with the presence of PrPres immunoreactivity. A few punctate PrPres deposits are seen at 120 dpi; a diffuse synaptic-like PrPRes immunostaining are found at 180dpi (z). Paraffin sections lightly counterstained with haematoxylin, A-P, bar = 25 μm; Q-Y, bar = 100 μm; Z, bar = 25 μm

**Figure 2.**
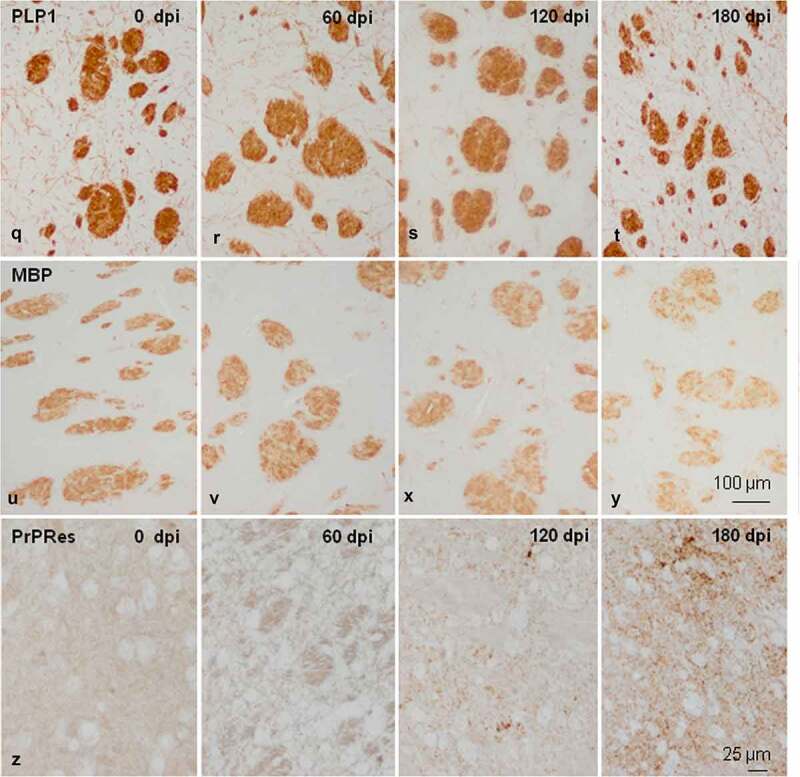
Continued

Two-way ANOVA also revealed a significant effect of sCJD MM1-inoculation for *gfap* [F [1,16] = 17.49 (P = 0.0006)] and *slc1a2* [F [1,16] = 9.008 (P = 0.0077)] gene expression. Bonferroni’s post-hoc test showed increased levels of *gfap* in inoculated animals at 160 dpi (P < 0.05), and 180 dpi (P < 0.05) when compared with age-matched non-inoculated mice. *slc1a2* mRNA levels were significantly increased in inoculated animals at 160 dpi (P < 0.05) when compared with non-inoculated mice.

In contrast to gene expression alterations in astrocytes, the expression of *olig1, cspg4* (encoding NG2), *plp1, mbp*, and *mct1* did not show modifications when compared with controls up to 180 dpi; the only significant interaction observed was between sCJD MM1-inoculation and the progression of the incubation time for *myrf* [F [2,16] = 4.295 (P = 0.03)] gene expression (Figure 1b). Bonferroni’s *post hoc* test indicated a significant increase in *myrf* expression levels at 180 dpi (P < 0.05) when compared to the control group.

Spongiform changes were first observed at 120 dpi in the middle layers of the cerebral cortex, plexiform layers of the hippocampus, and striatum. The number of vacuoles in the neuropil increased at 180 dpi in all the cerebral cortex, hippocampus, dentate gyrus, subiculum, thalamus, brain stem, cerebellum, and striatum.

**Figure 3.**
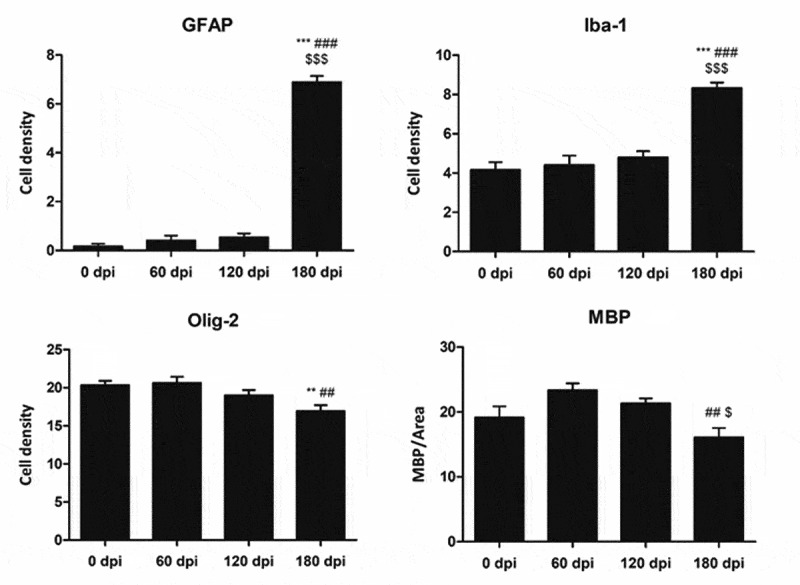
Quantification of GFAP-, Iba1- and Olig2-immunoreactive cells, and MBP densitometry (arbitrary units) per area at different dpi. Differences between inoculated groups at different incubation times are set at **p < 0.01, ***p < 0.001 vs. 0 dpi; ## p < 0.01, ### p < 0.001 vs. 60 dpi; and $ p < 0.05, $$$ p < 0.001 vs. 120 dpi

The morphological study was focused on the striatum because this was the most affected region in inoculated mice. Mild spongiform change occurred at 120 dpi and increased at 180 dpi (Figure 2a-d). A mild, non-significant increase in the number of astrocytes and microglial cells was noted at 120 dpi. A significant increase in the number of GFAP-immunoreactive astrocytes and Iba1-positive microglia appeared at 180 dpi (P = 0.000 and P = 0.000, respectively) (Figure 2e-l and Figure 3). The number of oligodendrocytes in the striatal fascicles showed a significant decrease at 180 dpi when compared to 0 and 60 dpi animals (P = 0.01 and P = 0.004, respectively) (Figure 2m-p and Figure 3). No differences in immunostaining of proteolipid protein 1 (PLP1) were observed with disease progression up to 180 dpi (Figure 2q-t and Figure 3). However, the immunoreactivity to myelin basic protein (MBP) focally decreased in the intrastriatal fascicles at 180 dpi when compared to 60 dpi (P = 0.002) and 120 dpi (P = 0.036) animals (Figure 2u-y and Figure 3). These alterations occurred in parallel with the recognition of PrP^Res^ immunostaining after inoculation. PrP^Res^ immunoreactivity was first seen at 120 dpi as fine focal punctuate dots, and increased at 180 dpi as diffuse synaptic-like PrP^Res^ deposits (Figure 2z).

## Discussion

The present results confirm the widely recognized involvement of astrocytes at early clinical stages of prion diseases, as revealed by the altered mRNA expression of many astrocyte markers at 160 dpi, and increased GFAP immunoreactivity at 180 dpi. The mRNA expression of oligodendroglial markers (olig1), adult oligodendroglial precursors (*csp4*), and genes encoding main myelin proteins *plp1, mbp*, and *mog* [16–20] are not altered with disease progression up to 180 dpi. Only the expression of myelin regulatory factor (*myrf*) which triggers myelination is significantly increased at 180 dpi, thus suggesting some compensatory function to balance initial at-risk myelination linked to the decrease in the number of oligodendrocytes at 180 dpi. PLP1 immunostaining is not altered, but a focal cotton-like decrease of MBP occurs in the intrastriatal fascicles at 180 dpi. These findings corroborate the relative resistance of the oligodendrocyte cell line in comparison with the reaction of astrocytes and microglia to PrP^SC^ in a validated model of human CJD. Nevertheless, our results also confirm that oligodendrocytes are vulnerable at advanced stages of murine CJD, as they have previously been noted to be in human sCJD [11–13].

Oligodendrocyte abnormalities may contribute to explain ultrastructural white matter myelin alterations in CJD, and other natural and experimentally induced animal prionopathies [11,21–23]. Moreover, oligodendrocyte alterations described in CJD [11–13] and present experimental model may link transition between mild to moderate white matter involvement in common forms compared with severe white matter involvement in panencephalopathic forms of CJD [24–28]. Although alterations of the white matter parallel the intensity of lesions of the grey matter in cases with long duration of the disease [28,29], pioneering panencephalopathic cases, described principally in Japan, were in favour of a primary alteration of the white matter [24,30,31]. Myelin and nerve fibre loss accompanied by variable astrocyte proliferation and phagocytosis of myelin debris is usually described in panencepaholopathic CJD, but, curiously, any information about oligodendrocytes in the white matter is almost disregarded. Nevertheless, engulfment of one or several oligodendrocytes by hypertrophic astrocytes (emperipolesis) was reported in five CJD cases with a long clinical course and devastated white matter [32]. Further studies are needed to elucidate astrocyte/oligodendrocyte interactions that sustain white matter alterations in CJD.
